# Peer support: A needs assessment for social support from trained peers in response to stress among medical physicists

**DOI:** 10.1002/acm2.12675

**Published:** 2019-07-29

**Authors:** Jennifer Johnson, Eric Ford, James Yu, Courtney Buckey, Shannon Fogh, Suzanne B. Evans

**Affiliations:** ^1^ Kelsey‐Seybold Clinic Houston TX USA; ^2^ Department of Radiation Oncology University of Washington Seattle WA USA; ^3^ Department of Radiation Oncology Yale University School of Medicine New Haven CT USA; ^4^ Department of Radiation Oncology Mayo Clinic Scottsdale AZ USA; ^5^ Department of Radiation Oncology University of California San Francisco CA USA

**Keywords:** burnout, patient safety, resilience, second victim, social support

## Abstract

**Purpose:**

Previous studies suggest that within radiation oncology, medical physicists (MP) experience high workloads. Little is known about how MPs use social support (SS) in times of stress.

**Methods:**

In collaboration with the Workgroup on Prevention of Medical Error, the American Association of Physicists in Medicine administered this Human Investigation Committee (HIC) approved email survey to 8566 members. Respondents were considered likely to seek SS if they answered (probably/definitely would) and unlikely to seek support if they answered (probably/definitely would not). Logistic regression was applied to determine associations between demographic factors and willingness to seek support as well as perception of barriers.

**Results:**

One thousand two hundred and ninety‐seven members (15.1%) accessed and gave consent for the survey. One thousand and one (11.7%) respondents answered all relevant questions. Respondents were predominantly male (69.1%), MP in radiation oncology (81.8%), private practice (51.6%), with practice duration> 10 yr (60.2%). MPs were likely to seek SS for personal physical illness (78.63%), involvement in a medical error (73.94%) or adverse patient outcome (75.17%). MPs sought SS in the setting of personal fatigue (33.2%) or burnout (44.3%). Barriers to seeking SS were lack of time (80.3%), and uncertainty about whom to access (70.7%). MPs responded that they would be most likely to seek support from an equally experienced medical physicist colleague (81.0%). Most MPs (67.0%) identified as having experienced stressors, with serious family illness (35.2%), or burnout (32.8%) being most common. Factors associated with MPs unwillingness to seek SS for medical error included> 20 yr in practice (vs still in training — OR 0.30, *P* = 0.015), and male gender (OR 0.60, *P* = 0.003). Male gender was associated with the lowest willingness to seek support (OR 2.10, *P* = 0.0001), but also with fewer perceived barriers (OR 1.60, *P* = 0.0075).

**Conclusion:**

Willingness to seek SS is demonstrated, and MPs want colleagues to provide support. Given these results, peer support could be considered among MPs.

## 1 INTRODUCTION

Among physicians, the provision of social support from trained peers in times of workplace‐associated stressors (e.g., burnout, medical error, or adverse patient events) has become a major movement,^1^, ^2^ initially launched by a landmark survey in 2012.^1^ Such a system has been not only highly valued by clinicians, but also cost effective for institutions.^3^, ^4^ As part of this, the physician realm has increasingly embraced programs aimed at providing peer support to their own, not just in the setting of medical error, but also in times of other crisis and stress both personal and professional. One example may include when the physician is experiencing fatigue, or the feeling of tiredness and decreased energy that results from prolonged mental or physical exertion, such as increased work intensity or long work hours. Another example is physician burnout, when he or she is experiencing the physical or mental collapse caused by overwork or stress; burnout syndrome is characterized by emotional exhaustion, cynicism, and reduced effectiveness that results in depersonalization and decreased personal accomplishment at work. Providing peer social support arose from the recognition that wellness is critical to high performance. Being involved in peer support in either role fulfills many of the facets of recovery and resilience after error or other stressors.

There is substantial rationale for believing that this paradigm may be also useful to medical physicists. Medical physicists are highly trained experts essential to the delivery of safe radiation therapy. The high risks involved in medical physics can create both acute and chronic stress, and studies of the relative task loads of the radiation oncology team indicate that the perceived task loads of medical physicists may be the highest among the various radiation oncology team members.^5^

Among physicians, the causes of burnout are well studied. These include chaotic work environments, clerical burden and inefficient workplaces,^6^ interference of work obligations with family events, lack of control over work schedule, medical error, and poor self‐care.^7^, ^8^ Among physicians, burnout has been linked to a list of untoward consequences, including lower patient satisfaction and lower quality of care, higher medical error rates and malpractice risk, higher physician and staff turnover, physician substance abuse, and even physician suicide.^9^, ^10^, ^11^, ^12^, ^13^, ^14^ While the drivers and consequences of burnout within medical physicists are not well characterized, many medical physicists also share these chaotic work environments. It is postulated that these challenging conditions exist within the work life of the medical physicist as well, and in addition to this high workload, may contribute to the 40% burnout rate of medical physicists.^15^, ^16^

In addition to these daily workplace‐associated causes of stress, clinicians and patient care team members are sometimes involved in a medical error, defined as a preventable adverse event affecting the patient.^17^ Although the career likelihood of being involved in a medical error for a medical physicist is unknown, a preventable medical error is a particularly devastating component of work stress impacting clinicians and patient care team members. While a patient is the first victim of, “an unanticipated adverse event, medical error or patient injury,”^1^ health care providers involved in such incidents are also traumatized, becoming second victims to such events.^19^, ^20^ The literature provides ample descriptions of the detrimental effects of harmful errors to providers as they suffer from the guilt of impacting a patient's morbidity, mortality and quality of life. In addition to guilt, providers also experience cognitive dissonance of the error or poor clinical outcome with self‐perceived infallibility, coupled with a life‐long commitment to do no harm.^21^ The second victim phenomenon has been studied extensively in the health system as a whole and is a recognized issue by the Agency for Healthcare Research (AHRQ)^18^ as well as the subject of an Institute for Healthcare Improvement (IHI) white paper.^22^ Within the Radiation Oncology community there is now an increased awareness of second victim and the need for social support, with the 2016 Canadian Winter School on Quality and Safety in Radiation Oncology^23^ and the 2016 American Society of Radiation Oncology (ASTRO) Annual Meeting both featuring presentations on support for clinicians in times of stress. Additionally, some academic radiation oncology departments (e.g., Johns Hopkins, Brigham and Women's Hospital, University of Missouri) can participate in their institution's peer support training.^2^

Although much has been learned among physicians regarding workplace‐associated stress and what promotes resiliency, there is no information about how we might best support medical physicists (e.g., from whom might this support might be acceptable? Is this support desirable to medical physics community? In what circumstances is this desirable?). The purpose of this study is to determine the willingness of medical physicists to access social support in times of stress, including medical error, as a first step toward understanding and addressing workplace‐associated stress.

## 2 MATERIALS AND METHODS

The Yale Human Investigation Committee (HIC) reviewed this study and determined it was exempt from review (HIC#1606017870). To help assess the support needs of medical physicists, the survey previously used in the physician community^1^ was minimally adapted for medical physicists and used with permission from the survey author. Through collaboration with the Professional Council of American Association of Physicists in Medicine (AAPM), the Working Group on the Prevention of Errors supported the distribution of this survey through email. The adapted survey questions were entered electronically into the survey instrument, and an invitation for participation was sent to the full membership of AAPM. Electronic informed consent was obtained. All responses were anonymized and only minimal demographic information was obtained through self‐report.

Medical physicists were surveyed general demographic questions regarding practice environment (predominantly academic practice, predominantly private practice, locums practice, residency); years of experience in the practice of medical physics (still in training, 0–5 yr, 6–10 yr, 11–20 yr, >20 yr); gender (male, female); age category (Under 30, 30–40, 41–50, 51–60, or over 60 yr old); and marital status (single, married, unmarried but in a committed relationship, divorced/ separated). Medical physicists were asked whether they would seek support within 14 predefined categories (Table 2), ranging from legal situations to medical error to interpersonal conflicts. Survey answers were categorized as support seeking vs nonsupport seeking. Support seeking category included responses of “probably would” or “definitely would” responses; nonsupport seeking included responses of “probably would not” or “definitely would not.” Survey respondents were categorized as seeking “any support” if they answered “probably would” or “definitely would” in response to any stressor. Medical physicists were then asked about perceived barriers to support, as well as from whom they felt comfortable seeking support (with the opportunity to free text responses for this item).

### 2.A Statistics

Using logistic regression, adjusting for demographic and practice categories, we analyzed whether a medical physicist would seek support for a medical error, as medical error is a particularly devastating stressor. Finally, we compared medical physicist rates of social support seeking to physicians per the report from Hu et al.^1^ using a Chi‐Square goodness of fit test using the physician reported percentage as the expected percentage.

### 2.B Results

The survey was sent to all 8566 members of the AAPM. One thousand four hundred and six members (16.4% response rate) accessed the survey, and 1297 of those (92.3%) gave consent for the survey. One thousand and one (11.7%) respondents answered all of the support‐related questions. Reflective of the AAPM membership demographics, respondents predominantly self‐identified as male (69.1%), as working primarily in radiation oncology (81.8%), as working in the private practice setting (51.6%), and as having been in practice either> 20 yr (36.4%) or 11–20 yr (23.8%). Detailed demographic information is shown in Table 1.

**Table 1:** Demographic characteristics of the medical physicist respondents with complete responses of the support survey

Variable	n(%)
Age (*n* = 1001)	
Under 30	65 (6.5%)
30–40	312 (31.1%)
41‐50	253 (25.2%)
51‐60	203 (20.3%)
Over 60	169 (16.9%)
Gender (*n* = 994)	
Female	307 (30.9%)
Male	687 (69.1%)
Marital status (*n* = 1001)	
Divorced/separated	37 (3.7%)
Married	804 (80.3%)
Single	107 (10.7%)
Unmarried but in a committed relationship	53 (5.3%)
Practice focus within medical physics (*n* = 1001)	
Therapeutic radiology/radiation oncology	819 (81.8%)
Nuclear medicine	6 (0.6%)
Diagnostic radiology	77 (7.7%)
Both diagnostic radiology and nuclear medicine	59 (5.9%)
Combination of the above	40 (4.0%)
Years in practice (*n* = 999)	
Still in training/residency	49 (4.9%)
0–5 yr	145 (14.5%)
6–10 yr	203 (20.3%)
11–20 yr	238 (23.8%)
More than 20 yr	364 (36.44%)

Medical physicist respondents were most likely to seek social support (Table 2) for a legal situation (80.5%), physical illness or mental illness in themselves (78.6% and 71.0%, respectively), physical illness or mental illness in a family member (72.5% and 69.0%, respectively), as well as after being involved in a medical error (74.0%) or adverse patient outcome (75.2%). They were not as willing to seek support for personal fatigue (33.2%) or burnout (44.3%). The top three identified barriers to seeking social support (Table 3) were lack of time (80.3%), uncertainty about whom to access for support (70.7%), and confidentiality concerns (68.4%).

**Table 2:** The perceived *situations which might warrant social support* identified by medical physicist respondents

Situation which might warrant social support	Percent of medical physicists willing to seek social support for the situation
Legal situation	80.5%
Physical illness in self	78.6%
Adverse patient outcome^a^	75.2%
Substance abuse	74.2%
Medical error^b^	74.0%
Physical illness in family member	72.5%
Mental illness in self	71.0%
Mental illness in family member	69.0%
Interpersonal conflict in the workplace	65.9%
Poor patient outcome, regardless of responsibility	52.2%
Personal life struggle	47.7%
Personal burnout^c^	44.3%
Personal fatigue^d^	33.2%
Interpersonal conflict outside the workplace	25%

a Adverse patient outcome — a poor outcome for the patient, which may be related to their underlying disease, a known potential complication to the treatment or procedure, or a suboptimal care process.

b Medical error — a preventable adverse patient event.

c Burnout — physical or mental collapse caused by overwork or stress; syndrome characterized by emotional exhaustion, cynicism, and reduced effectiveness that results in depersonalization and decreased personal accomplishment at work.

d Fatigue — the feeling of tiredness and decreased energy that results from prolonged mental or physical exertion, such as increased work intensity or long work hours.

**Table 3:** The perceived *barriers to seeking social support* identified by medical physicist respondents

Barrier to seeking social support	Prevalence
Lack of time	80.3%
Uncertainty about who to access for support	70.7%
Confidentiality concerns	68.4%
Negative career impact	64.3%
Unwanted documentation on one's record	63.1%
Concern about unwanted intervention	61.1%
Difficulty with access to services	61.1%
Stigma of mental health care	54.4%
Cost	54.3%
Fear of legal consequences	52.2%
Feeling that my problems are not important	48.0%
Concern that no one will understand my problems	45.4%
Feeling that “using services means that I am weak”	40.0%

Medical physicist respondents indicated that they would be most likely to seek support (Table 4) from a significant other or spouse (89.0%), or a medical physicist colleague of at least equal experience trained in peer support (69.2% and 62.6% for an external colleague and co‐worker, respectively). They were least likely to seek support from a nursing colleague (30.1%), radiation therapist (27.5%), or a medical physicist colleague of lesser experience (22.9%). Approximately two‐thirds of respondents (67.0%) reported as having been exposed to at least one of the following stressors: serious family illness (35.2%), frequent or constant burnout (32.8%), death of a family member (24.9%), adverse patient event (14.2%), personal mental illness (9.5%), or desire for self‐harm (4.1%). An additional 5.3% remarked that they had been through another personal crisis (e.g., divorce or loss of a romantic partner, bankruptcy, challenges related to pregnancy and child rearing, workplace bullying, or job loss). Of the respondents who had exposure to such stressors, 62.7% of them sought some form of social support. Only 28.9% reported that they did not experience burnout to any degree.

**Table 4:** The *potential source of social support* identified by a medical physicist respondent when faced with stressful situations and percent of medical physicists willing to seek support from each

Potential source of social support	Percent of medical physicists willing to seek social support from potential source
Spouse or significant other	89.0%
Physicist colleague trained in peer support from outside one's workplace	69.2%
Physicist colleague trained in peer support from one's workplace	62.6%
Mental health professional	57.9%
Departmental authority figure	56.5%
Employee sponsored assistance program	56.3%
Radiation oncologist or radiologist colleague	51.9%
Medical dosimetrist	43.2%
Clergy member	32.6%
Nurse colleague	30.1%
Radiation therapist	27.5%
Junior physicist colleague	22.9%

**Figure 1:**
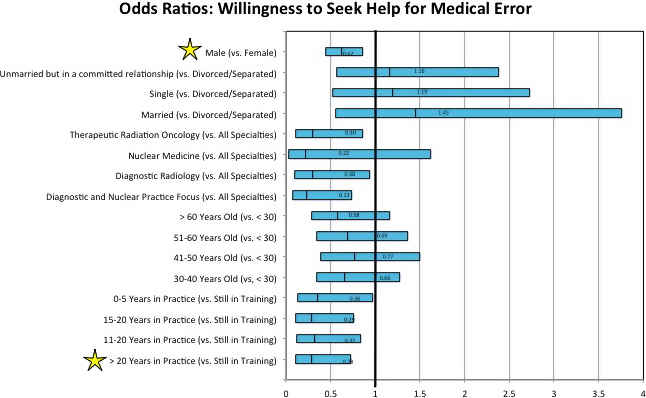
Odds ratios to determine demographic predictors of medical physicist willingness to seek social support after a medical error calculated by using logistic regression in this study. An odds ratio represents the odds that an outcome (e.g., a medical physicist would seek social support) will occur given a particular exposure (e.g., a medical error), compared to the odds of the outcome occurring (e.g., a medical physicist would seek social support) in the absence of the exposure (e.g., a medical error). Only two demographic predictors were associated with lower odds of outcome compared to the baseline category. Medical physicists with> 20 yr in practice (vs medical physicists still in training) were unwilling to seek social support after a medical error (OR 0.30, *P* = 0.015). Also, medical physicists of male gender (vs female) were unwilling to seek social support after a medical error (OR 0.60, *P* = 0.003). The vertical line represents an OR of 1.0

Odds ratios calculated from the logistic regression to determine demographic predictors of medical physicist willingness to seek social support after a medical error only resulted in two risk factors for unwillingness to seek social support. An odds ratio represents the odds that an outcome (e.g., a medical physicist would seek social support) will occur given a particular exposure (e.g., a medical error), compared to the odds of the outcome occurring (e.g., a medical physicist would seek social support) in the absence of the exposure (e.g., a medical error). For a medical error exposure, only two demographic predictors were associated with lower odds of outcome compared to the baseline category, as depicted in Figure 1. Medical physicists with> 20 yr in practice (vs. medical physicists still in training) were unwilling to seek social support after a medical error (OR 0.30, *P* = 0.015). Also, medical physicists of male gender (vs female) were unwilling to seek social support after a medical error (OR 0.60, *P* = 0.003). Interestingly, results from the logistic regression did not find any demographic factors associated with unwillingness to seek support for burnout. Medical physicist respondents were found to be more likely than their physician colleagues to seek social support in all surveyed scenarios (Table 5).

**Table 5:** Medical physicists (MP) results were compared to historical data on physicians (MD, where available^1^) for willingness to seek social support using chi squared goodness‐of‐fit

Situation which might warrant social support	Percent willing to seek social support for the situation		*P*
	MP	MD	
Personal physical illness	79%	62%	<0.001
Personal mental illness	71%	50%	<0.001
Involvement with an adverse patient outcome	75%	63%	<0.001
Involvement with a medical error	74%	67%	<0.001
Awareness of an adverse patient outcome	52%	38%	<0.001
Personal fatigue	33%	9%	<0.001
Personal burnout	44%	24%	<0.001

## 3 DISCUSSION

This study demonstrated that medical physicists who responded to the support survey are willing to seek social support in times of stress or following a medical error (Table 2). Additionally, the majority of the survey respondents admitted to having at least one stressful event in their life over the prior year. Although one's spouse or significant other was the dominant source of support, a spouse may not have the tools to adequately provide the most effective support. The next most popular group was a medical physicist colleague at a different institution trained in peer support, suggesting that a peer who knows the stresses of the job — but is somewhat distant to oneself or the stressful situation at hand — makes the ideal peer supporter (provided they have the requisite training^2^ in providing peer support.) Followed closely behind this was a local medical physicist colleague also trained in providing peer support. Interestingly, the next most popular response was a mental health professional, suggesting that support expertise was valued compared to seeking support in general from other work colleagues or sponsored assistance program.

This is, to our knowledge, the first study of its kind that examines the willingness of medical physicists to seek social support. Similar trends are found between physicians^1^ and medical physicists with respect to which events would trigger the individual to seek peer support. The willingness to seek support in response to stress from our survey respondents often exceeds physician results,^1^ which suggests that medical physicists could benefit from the implementation of peer support programs as physicians have.^1^, ^2^ Of note, as only about half of medical physicists would feel comfortable receiving peer support from their physician colleagues, it seems unlikely that support groups for the two professional groups could be combined. This is notable, because for institutions with small numbers of medical physicists, being able to access peer support in collaboration with other institutions or national organizations may be particularly important.

This present study complements prior work done on the topic of burnout, as the reported rates of frequent or constant burnout are similar.^16^ Burnout appears to be a problematic area for peer support. In this survey the majority of respondents would not seek social support for burnout. Perhaps it is not widely known that peer support for burnout^24^ has benefits, with improvements seen in sense of belonging, behavior change, and self‐confidence among those receiving peer support. The survey respondents also disclosed a variety of events that might lead them to seek peer support that extends beyond the workplace (e.g., parenting challenges including infertility, new childbirth, or miscarriage; toxic work environments, or loss of a beloved pet.) The surveyed group drew attention through free text responses to the fact that stress accompanies not only acute events, but also chronic events such as caring for an aging parent, workplace bullying, and new parenthood.

Limitations of this study include sample size and the associated self‐selection bias. As such, the respondents who chose to participate in the study may not represent the medical physics population as a whole, so the survey results may not have the generalizability of the full AAPM membership. Social desirability, or the tendency to respond to personal or socially sensitive content in a socially acceptable direction (e.g., respondents willingness to say that they were involved in a medical error, even anonymously), may also bias and limit the interpretability of the survey results. This survey material is quite sensitive, and some people did decline to participate based on privacy concerns. However, this indicates that the data on incidence of stressors are more likely to be underestimated, highlighting the importance of this work. Finally, it is not clear how best to use this information. Since the majority of the respondents indicate that they would be most likely to seek peer support from an individual outside of their home institution who was trained in peer support, it may be best to develop a peer support program through the AAPM. The logistics of how to administer such a program are ripe for exploration.

This survey was able to demonstrate a substantial willingness of medical physicists to seek social support in response to a large variety of stressful situations. Medical physicist colleagues trained in peer support, particularly those from another institution, were favored as the providers of this support. It seems that a centralized peer support system, perhaps through a national organization that maintains confidentiality in some way, could be a successful strategy.

## CONFLICT OF INTEREST

The authors have no relevant conflict of interest to disclose.

## Supporting information


**Table S1**. Survey Instrument, as constructed.
