# Supplementary material for: Peer support: A needs assessment for social support from trained peers in response to stress among medical physicists
Source: J Appl Clin Med Phys. 2019 Jul 29;20(9):157–62. doi: 10.1002/acm2.12675 (PMC6753865; doi:10.1002/acm2.12675)
Supplement: Supplementary file 1 — Table S1. Survey Instrument, as constructed. [file ACM2-20-157-s001.pdf]

Medical physics training and practice can be stressful. In order to assess the support mechanisms currently in place across the nation, we appreciate your participation in this confidential, anonymous survey (49 questions).

Support mechanisms are those people or services one would turn to in a stressful situation.

Most medical physicists encounter one or more of the following stressful situations at some point in their lives. For each, please indicate whether you would or would not seek support for the following difficulties:

*** 1. Personal Life struggles**

○ Definitely would not ○ Probably would not ○ Probably would ○ Definitely would

*** 2. Physical illness in yourself**

○ Definitely would not ○ Probably would not ○ Probably would ○ Definitely would

*** 3. Physical illness in a family member**

○ Definitely would not ○ Probably would not ○ Probably would ○ Definitely would

*** 4. Mental health illness in yourself**

○ Definitely would not ○ Probably would not ○ Probably would ○ Definitely would

*** 5. Mental health illness in a family member**

○ Definitely would not ○ Probably would not ○ Probably would ○ Definitely would

*** 6. Medical error that you were involved with**

○ Definitely would not ○ Probably would not ○ Probably would ○ Definitely would

*** 7. Adverse patient event that you were involved with**

Definitely would not Probably would not Probably would Definitely would

*** 8. Poor patient outcome regardless of your responsibility**

Definitely would not Probably would not Probably would Definitely would

*** 9. Interpersonal conflict in the workplace**

Definitely would not Probably would not Probably would Definitely would

*** 10. Interpersonal conflict outside of the workplace**

Definitely would not Probably would not Probably would Definitely would

*** 11. Personal Fatigue**

Definitely would not Probably would not Probably would Definitely would

*** 12. Personal Burnout**

Definitely would not Probably would not Probably would Definitely would

*** 13. Legal Situation**

Definitely would not Probably would not Probably would Definitely would

*** 14. Substance abuse**

Definitely would not Probably would not Probably would Definitely would

Next

Powered by

SurveyMonkey®

See how easy it is to create a survey.

Medical physics training and practice can be stressful. In order to assess the support mechanisms currently in place across the nation, we appreciate your participation in this confidential, anonymous survey (49 questions).

If you ever wanted to seek support, please indicate whether you agree or disagree that the following factors would be a barrier:

*** 15. Lack of time**

○ strongly disagree ○ somewhat disagree ○ somewhat agree ○ strongly agree

*** 16. Concerns about confidentiality**

○ strongly disagree ○ somewhat disagree ○ somewhat agree ○ strongly agree

*** 17. Concern that "nobody will be able to understand my problems"**

○ strongly disagree ○ somewhat disagree ○ somewhat agree ○ strongly agree

*** 18. Stigma of mental health care**

○ strongly disagree ○ somewhat disagree ○ somewhat agree ○ strongly agree

*** 19. Feeling that "using services means that I am weak"**

○ strongly disagree ○ somewhat disagree ○ somewhat agree ○ strongly agree

*** 20. Cost**

○ strongly disagree ○ somewhat disagree ○ somewhat agree ○ strongly agree

*** 21. Feeling that my problems are not important**

○ strongly disagree ○ somewhat disagree ○ somewhat agree ○ strongly agree

*** 22. Fear of unwanted intervention**

- ◯ strongly disagree ◯ somewhat disagree ◯ somewhat agree ◯ strongly agree

*** 23. Difficulty accessing services (e.g., feeling that support services are not easily available)**

- ◯ strongly disagree ◯ somewhat disagree ◯ somewhat agree ◯ strongly agree

*** 24. Not knowing who to go to**

- ◯ strongly disagree ◯ somewhat disagree ◯ somewhat agree ◯ strongly agree

*** 25. Fear of legal consequences**

- ◯ strongly disagree ◯ somewhat disagree ◯ somewhat agree ◯ strongly agree

*** 26. Fear of documentation on my record**

- ◯ strongly disagree ◯ somewhat disagree ◯ somewhat agree ◯ strongly agree

*** 27. Fear of negative impact on my career**

- ◯ strongly disagree ◯ somewhat disagree ◯ somewhat agree ◯ strongly agree

Prev Next

Powered by

SurveyMonkey®

See how easy it is to create a survey.

Please state the likelihood that you would or would not seek support from each of the following when faced with stressful situations:

*** 28. An Employee sponsored assistance program**

- ◯ would not seek support
- ◯ not very likely to seek support
- ◯ somewhat likely to seek support
- ◯ very likely to seek support

*** 29. Graduate Medical Education Director**

- ◯ would not seek support
- ◯ not very likely to seek support
- ◯ somewhat likely to seek support
- ◯ very likely to seek support

*** 30. Psychiatrist or other mental health professional**

- ◯ would not seek support
- ◯ not very likely to seek support
- ◯ somewhat likely to seek support
- ◯ very likely to seek support

*** 31. Clergy member**

- ◯ would not seek support

- not very likely to seek support
- somewhat likely to seek support
- very likely to seek support

*** 32. Department or section Chair or Vice Chair**

- would not seek support
- not very likely to seek support
- somewhat likely to seek support
- very likely to seek support

*** 33. Faculty trained in peer support**

- would not seek support
- not very likely to seek support
- somewhat likely to seek support
- very likely to seek support

*** 34. Faculty colleague**

- would not seek support
- not very likely to seek support
- somewhat likely to seek support
- very likely to seek support

*** 35. Chief or senior resident of your program**

- would not seek support
- not very likely to seek support
- somewhat likely to seek support
- very likely to seek support

* 36. Resident trained in peer support

- ◯ would not seek support
- ◯ not very likely to seek support
- ◯ somewhat likely to seek support
- ◯ very likely to seek support

* 37. Resident colleague

- ◯ would not seek support
- ◯ not very likely to seek support
- ◯ somewhat likely to seek support
- ◯ very likely to seek support

38. Other?

- ◯ yes
- ◯ no

Other (please specify)

[ ]

Prev Next

Powered by
SurveyMonkey®
See how easy it is to create a survey.

In the past year, have you experienced any of the following?

*** 39. Serious adverse event involving a patient for whom you were caring at the time**

◯ Yes ◯ No

*** 40. Serious personal physical illness**

◯ Yes ◯ No

*** 41. Personal mental illness**

◯ Yes ◯ No

*** 42. Feelings of wanting to harm yourself**

◯ Yes ◯ No

*** 43. Serious illness among family (parents, significant other, brothers/sisters, children)**

◯ Yes ◯ No

*** 44. Death in the family**

◯ Yes ◯ No

*** 45. Other personal crisis (e.g., divorce, bankruptcy, etc)**

◯ Yes ◯ No

*** 46. Other (please specify)**

◯ Yes ◯ No

Other (please specify)

* **47. If you have experienced any of these, please specify what sort of services you sought, if any.**

◯ Yes, I have experienced one of these ◯ No, I have not experienced one of these

The type of support I sought was:

Prev Next

Powered by

SurveyMonkey®

See how easy it is to create a survey.

Medical physics training and practice can be stressful. In order to assess the support mechanisms currently in place across the nation, we appreciate your participation in this confidential, anonymous survey (49 questions).

Last page! Thank you. #likeaboss

Background

*** 48. Which of the following best describes you?**

- ◯ Medical Physicist in an academic based practice
- ◯ Medical Physicist in a private based practice
- ◯ Medical Physics resident

Other (please specify)

49. If you are a medical physics resident, what year are you?

- ◯ 1st year
- ◯ 2nd year
- ◯ I am not a resident

Prev | Done

Powered by
SurveyMonkey®
See how easy it is to create a survey.
